# *tert*-Butyl 4-(2-diazo­acet­yl)piperazine-1-carboxyl­ate

**DOI:** 10.1107/S1600536810016211

**Published:** 2010-05-08

**Authors:** Åsmund Kaupang, Carl Henrik Görbitz, Tore Hansen

**Affiliations:** a Department of Chemistry, University of Oslo, PO Box 1033 Blindern, N-0315 Oslo, Norway

## Abstract

The title crystal structure, C_11_H_18_N_4_O_3_, is the first diazo­acetamide in which the diazo­acetyl group is attached to an N atom. The piperazine ring is in a chair form and hence the mol­ecule has an extended conformation. Both ring N atoms are bonded in an essentially planar configuration with the sum of the C—N—C angles being 359.8 (2) and 357.7 (2)°. In the crystal structure, the O atom of the diazo­acetyl group accepts two H atoms from C—H donors, thus generating chains of weak hydrogen-bonded *R*_2_^1^(7) rings.

## Related literature

For the only other reported synthesis of a diazo­acetamide in the Chemical Abstracts Service (CAS, American Chemical Society, 2008 ▶) with a 1,4-diaza six-membered ring, see: Mickelson *et al.* (1996 ▶). For other diazo­acetamides, see: Ouihia *et al.* (1993 ▶). For related structures, see: Fenlon *et al.* (2007 ▶); Wang *et al.* (2006 ▶); Miller *et al.* (1991 ▶). For synthetic details, see: Kaupang (2010 ▶); Toma *et al.* (2007 ▶). For hydrogen-bond graph-set notation, see: Bernstein *et al.* (1995 ▶). For a description of the Cambridge Structural Database, see: Allen (2002 ▶).
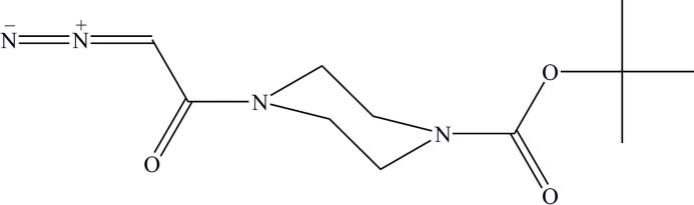


## Experimental

### 

#### Crystal data

C_11_H_18_N_4_O_3_
*M* *_r_* = 254.29
Monoclinic, 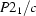

*a* = 14.654 (10) Å
*b* = 10.548 (7) Å
*c* = 8.553 (6) Å
β = 91.122 (6)°
*V* = 1321.8 (15) Å^3^
*Z* = 4
Mo *K*α radiation
μ = 0.10 mm^−1^
*T* = 105 K
0.55 × 0.42 × 0.08 mm

#### Data collection

Bruker APEXII CCD diffractometer
Absorption correction: multi-scan (*SADABS*; Bruker, 2007 ▶) *T*_min_ = 0.870, *T*_max_ = 0.992
7308 measured reflections
2692 independent reflections
2111 reflections with *I* > 2σ(*I*)
*R*_int_ = 0.041

#### Refinement

*R*[*F* ^2^ > 2σ(*F* ^2^)] = 0.038
*wR*(*F* ^2^) = 0.103
*S* = 1.03
2692 reflections
169 parameters
H atoms treated by a mixture of independent and constrained refinement
Δρ_max_ = 0.17 e Å^−3^
Δρ_min_ = −0.23 e Å^−3^

### 

Data collection: *APEX2* (Bruker, 2007 ▶); cell refinement: *SAINT-Plus* (Bruker, 2007 ▶); data reduction: *SAINT-Plus*; program(s) used to solve structure: *SHELXTL* (Sheldrick, 2008 ▶); program(s) used to refine structure: *SHELXTL*; molecular graphics: *SHELXTL*; software used to prepare material for publication: *SHELXTL*.

## Supplementary Material

Crystal structure: contains datablocks I, global. DOI: 10.1107/S1600536810016211/lh5033sup1.cif

Structure factors: contains datablocks I. DOI: 10.1107/S1600536810016211/lh5033Isup2.hkl

Additional supplementary materials:  crystallographic information; 3D view; checkCIF report

## Figures and Tables

**Table 1:** Hydrogen-bond geometry (Å, °)

*D*—H⋯*A*	*D*—H	H⋯*A*	*D*⋯*A*	*D*—H⋯*A*
C1—H1⋯O1^i^	0.946 (17)	2.313 (18)	3.250 (3)	170.6 (14)
C3—H32⋯O1^i^	0.99	2.36	3.327 (3)	164

Symmetry code: (i) 
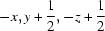
.

**Fig. 1.**
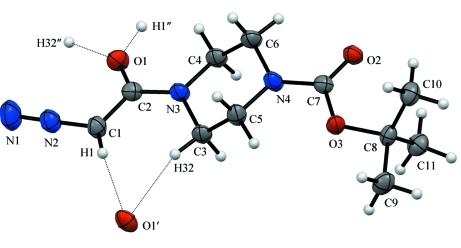
The asymmetric unit of (I), with atomic numbering indicated, together with selected atoms of neighbouring molecules connected by weak hydrogen bonds shown as dotted lines (see Table 1, symmetry operations are -x,1/2+y,1/2-z for O', -x,-1/2+y,1/2-z for H1" and H32"). Displacement ellipsoids are shown at the 50% probability level with H atoms as spheres of arbitrary size.

## supplementary crystallographic information

### Comment

The tert-butyl 4-(2-diazoacetyl)piperazine-1-carboxylate (I) was prepared as part of a series of diazoacetamides, to be used in intramolecular C—H insertion reactions (Kaupang, 2010). It was synthesized from tert-butyl 4-(2-bromoacetyl)piperazine-1-carboxylate by a slight modification of the procedure reported by Toma *et al.* (2007), employing 1,1,3,3-tetramethylguanidine as the base instead of 1,8-diazabicyclo[5.4.0]undec-7-ene.

Diffraction data were first collected at ambient temperature, yielding a structure with a massively disordered six-membered ring. At 105 K the ring is completely ordered in a well defined chair conformation, Fig. 1.

The diazoacetyl moiety is not an uncommon functional group in organic molecules, however only 15 occurrences were found in the Cambridge Structural Database (Version 5.31 of November 2009; Allen, 2002), and none where, as here, the group is attached to a N atom. In only two structures the group sits on a non-aromatic ring (Miller *et al.*, 1991; Fenlon *et al.*, 2007).

In a model molecule like trimethylamine the N atom is located about 0.45 Å above the plane defined by the three C atoms. In the structure of (I) N3 is 0.125 (2) Å above the plane defined by C2, C3 and C4, while N4 is only 0.039 (2) Å above the plane defined by C5, C6 and C7, which essentially shows a planar configuration (sum of C—N—C angles 359.8 (2) °). This is due to the double bond character of the amidic N3—C2 and N4—C7 bonds measuring 1.3515 (18) and 1.3483 (18) Å, respectively. An example of a related structure is 1,4-di(chloroacetyl)piperazine (Wang *et al.*, 2006).

In the crystal structure, the O atom of the diazoacetyl group accepts two H atoms from C—H donors, thus generating chains of hydrogen-bonded R^1^_2_(7) rings (Bernstein *et al.*,  1995).

### Experimental

A 2.5 ml vial containing tert-butyl 4-(2-diazoacetyl)piperazine-1-carboxylate (10.8 mg) and dichloromethane (1000 ml) was capped and a pinhole (0.5 mm) was made in the cap to allow for vapour diffusion of solvents. This vial was placed inside a 25 ml vial containing n-pentane (8 ml) that was subsequently capped and stored in the dark at ambient temperature for approximately 48 hours, affording bright yellow plate-shaped crystals.

### Refinement

Coordinates were refined for H1 (bonded to C1), which is involved in the shortest intermolcular interaction. Other H atoms were positioned with idealized geometry and fixed C–H distances set to 0.98 Å (methyl) or 0.99 Å (methylene). Free rotation was permitted for the methyl groups. U_iso_ values were 1.2U_eq_ of the carrier atom or 1.5U_eq_ for methyl groups.

### Crystal data

**Table:**

C_11_H_18_N_4_O_3_	*F*(000) = 544
*M**_r_* = 254.29	*D*_x_ = 1.278 Mg m^−^^3^
Monoclinic, *P*2_1_/*c*	Mo *K*α radiation, λ = 0.71073 Å
*a* = 14.654 (10) Å	Cell parameters from 2404 reflections
*b* = 10.548 (7) Å	θ = 2.4–26.4°
*c* = 8.553 (6) Å	µ = 0.10 mm^−^^1^
β = 91.122 (6)°	*T* = 105 K
*V* = 1321.8 (15) Å^3^	Plate, yellow
*Z* = 4	0.55 × 0.42 × 0.08 mm

### Data collection

**Table:**

Bruker APEXII CCD diffractometer	2692 independent reflections
Radiation source: fine-focus sealed tube	2111 reflections with *I* > 2σ(*I*)
graphite	*R*_int_ = 0.041
Detector resolution: 8.3 pixels mm^-1^	θ_max_ = 26.4°, θ_min_ = 2.4°
Three sets of frames each taken over 0.3° ω rotation with 20 s exposure time. Detector set at 2θ = 26°, crystal–to–detector distance 6.00 cm. scans	*h* = −17→18
Absorption correction: multi-scan (*SADABS*; Bruker, 2007)	*k* = −10→13
*T*_min_ = 0.870, *T*_max_ = 0.992	*l* = −10→10
7308 measured reflections	

### Refinement

**Table:**

Refinement on *F*^2^	Primary atom site location: structure-invariant direct methods
Least-squares matrix: full	Secondary atom site location: difference Fourier map
*R*[*F*^2^ > 2σ(*F*^2^)] = 0.038	Hydrogen site location: inferred from neighbouring sites
*wR*(*F*^2^) = 0.103	H atoms treated by a mixture of independent and constrained refinement
*S* = 1.03	*w* = 1/[σ^2^(*F*_o_^2^) + (0.0463*P*)^2^ + 0.1671*P*] where *P* = (*F*_o_^2^ + 2*F*_c_^2^)/3
2692 reflections	(Δ/σ)_max_ = 0.001
169 parameters	Δρ_max_ = 0.17 e Å^−^^3^
0 restraints	Δρ_min_ = −0.22 e Å^−^^3^

### Special details

**Table:**

Experimental. Crystallized from dichloromethane and n-pentane.
Geometry. All esds (except the esd in the dihedral angle between two l.s. planes) are estimated using the full covariance matrix. The cell esds are taken into account individually in the estimation of esds in distances, angles and torsion angles; correlations between esds in cell parameters are only used when they are defined by crystal symmetry. An approximate (isotropic) treatment of cell esds is used for estimating esds involving l.s. planes.
Refinement. Refinement of *F*^2^ against ALL reflections.

### Fractional atomic coordinates and isotropic or equivalent isotropic displacement parameters (Å^2^)

**Table:**

	*x*	*y*	*z*	*U*_iso_*/*U*_eq_	
O1	0.02567 (7)	0.20344 (9)	0.19361 (12)	0.0353 (3)	
O2	0.38950 (6)	0.35633 (9)	0.62995 (11)	0.0296 (2)	
O3	0.33388 (6)	0.55670 (8)	0.64091 (11)	0.0294 (2)	
N1	−0.14125 (10)	0.34028 (15)	−0.00383 (19)	0.0562 (4)	
N2	−0.08817 (8)	0.37402 (12)	0.08229 (16)	0.0377 (3)	
N3	0.10933 (7)	0.34931 (10)	0.32642 (14)	0.0293 (3)	
N4	0.27460 (8)	0.42179 (11)	0.46913 (15)	0.0350 (3)	
C1	−0.02502 (10)	0.41229 (14)	0.18201 (17)	0.0324 (3)	
H1	−0.0254 (10)	0.4999 (17)	0.2058 (19)	0.039*	
C2	0.03721 (9)	0.31392 (13)	0.23569 (16)	0.0276 (3)	
C3	0.11676 (9)	0.47080 (12)	0.40835 (18)	0.0301 (3)	
H31	0.0971	0.4604	0.5177	0.036*	
H32	0.0760	0.5337	0.3568	0.036*	
C4	0.17261 (10)	0.25284 (13)	0.38538 (18)	0.0317 (3)	
H41	0.1681	0.1763	0.3188	0.038*	
H42	0.1561	0.2287	0.4931	0.038*	
C5	0.21342 (9)	0.51824 (13)	0.40729 (18)	0.0322 (3)	
H51	0.2304	0.5397	0.2990	0.039*	
H52	0.2188	0.5960	0.4717	0.039*	
C6	0.26896 (10)	0.30251 (13)	0.38538 (19)	0.0349 (4)	
H61	0.3104	0.2399	0.4357	0.042*	
H62	0.2886	0.3150	0.2763	0.042*	
C7	0.33775 (9)	0.43825 (12)	0.58409 (16)	0.0259 (3)	
C8	0.39795 (9)	0.59837 (13)	0.76528 (15)	0.0275 (3)	
C9	0.36780 (11)	0.73381 (14)	0.79087 (18)	0.0387 (4)	
H91	0.3721	0.7811	0.6927	0.058*	
H92	0.4073	0.7734	0.8707	0.058*	
H93	0.3045	0.7346	0.8258	0.058*	
C10	0.49473 (10)	0.59327 (15)	0.70952 (17)	0.0354 (4)	
H101	0.4989	0.6386	0.6099	0.053*	
H102	0.5129	0.5047	0.6951	0.053*	
H103	0.5354	0.6333	0.7873	0.053*	
C11	0.38403 (10)	0.52052 (14)	0.91124 (17)	0.0351 (4)	
H111	0.3190	0.5190	0.9358	0.053*	
H112	0.4186	0.5583	0.9987	0.053*	
H113	0.4055	0.4337	0.8939	0.053*	

### Atomic displacement parameters (Å^2^)

**Table:**

	*U*^11^	*U*^22^	*U*^33^	*U*^12^	*U*^13^	*U*^23^
O1	0.0331 (6)	0.0239 (5)	0.0487 (6)	−0.0044 (4)	−0.0025 (5)	−0.0072 (5)
O2	0.0298 (5)	0.0242 (5)	0.0348 (5)	0.0047 (4)	−0.0017 (4)	0.0004 (4)
O3	0.0326 (5)	0.0217 (5)	0.0336 (5)	0.0019 (4)	−0.0074 (4)	−0.0042 (4)
N1	0.0446 (8)	0.0565 (10)	0.0665 (10)	−0.0058 (7)	−0.0207 (8)	−0.0054 (8)
N2	0.0320 (7)	0.0341 (7)	0.0469 (8)	−0.0010 (5)	−0.0060 (6)	−0.0007 (6)
N3	0.0279 (6)	0.0200 (6)	0.0398 (7)	0.0018 (5)	−0.0042 (5)	−0.0041 (5)
N4	0.0304 (6)	0.0239 (6)	0.0503 (8)	0.0057 (5)	−0.0125 (6)	−0.0096 (5)
C1	0.0297 (8)	0.0281 (8)	0.0390 (8)	−0.0023 (6)	−0.0063 (6)	−0.0020 (6)
C2	0.0257 (7)	0.0241 (7)	0.0332 (7)	−0.0038 (5)	0.0042 (6)	−0.0018 (6)
C3	0.0324 (8)	0.0204 (7)	0.0373 (8)	0.0039 (6)	−0.0050 (6)	−0.0041 (6)
C4	0.0342 (8)	0.0202 (7)	0.0406 (8)	0.0031 (6)	−0.0035 (6)	−0.0040 (6)
C5	0.0341 (8)	0.0224 (7)	0.0396 (8)	−0.0002 (6)	−0.0089 (6)	−0.0014 (6)
C6	0.0325 (8)	0.0264 (7)	0.0455 (9)	0.0053 (6)	−0.0054 (7)	−0.0121 (6)
C7	0.0241 (7)	0.0237 (7)	0.0300 (7)	−0.0013 (5)	0.0030 (5)	−0.0008 (6)
C8	0.0314 (7)	0.0259 (7)	0.0249 (7)	−0.0041 (6)	−0.0037 (6)	−0.0013 (5)
C9	0.0523 (10)	0.0268 (8)	0.0367 (8)	−0.0006 (7)	−0.0048 (7)	−0.0049 (6)
C10	0.0346 (8)	0.0390 (8)	0.0325 (8)	−0.0078 (7)	−0.0006 (6)	0.0005 (6)
C11	0.0398 (8)	0.0354 (8)	0.0302 (8)	−0.0032 (7)	0.0025 (6)	0.0019 (6)

### Geometric parameters (Å, °)

**Table:**

O1—C2	1.2303 (17)	C4—H41	0.9900
O2—C7	1.2099 (16)	C4—H42	0.9900
O3—C7	1.3422 (17)	C5—H51	0.9900
O3—C8	1.4722 (16)	C5—H52	0.9900
N1—N2	1.1189 (18)	C6—H61	0.9900
N2—C1	1.3099 (19)	C6—H62	0.9900
N3—C2	1.3515 (18)	C8—C10	1.506 (2)
N3—C4	1.4600 (18)	C8—C11	1.511 (2)
N3—C3	1.4635 (18)	C8—C9	1.513 (2)
N4—C7	1.3483 (18)	C9—H91	0.9800
N4—C5	1.4490 (18)	C9—H92	0.9800
N4—C6	1.4494 (19)	C9—H93	0.9800
C1—C2	1.450 (2)	C10—H101	0.9800
C1—H1	0.946 (17)	C10—H102	0.9800
C3—C5	1.503 (2)	C10—H103	0.9800
C3—H31	0.9900	C11—H111	0.9800
C3—H32	0.9900	C11—H112	0.9800
C4—C6	1.506 (2)	C11—H113	0.9800
			
C7—O3—C8	120.57 (10)	N4—C6—H61	109.6
N1—N2—C1	179.04 (18)	C4—C6—H61	109.6
C2—N3—C4	119.35 (12)	N4—C6—H62	109.6
C2—N3—C3	124.53 (11)	C4—C6—H62	109.6
C4—N3—C3	113.83 (11)	H61—C6—H62	108.1
C7—N4—C5	125.93 (12)	O2—C7—O3	125.31 (12)
C7—N4—C6	120.26 (11)	O2—C7—N4	124.15 (13)
C5—N4—C6	113.59 (12)	O3—C7—N4	110.52 (11)
N2—C1—C2	114.68 (13)	O3—C8—C10	110.59 (12)
N2—C1—H1	115.7 (9)	O3—C8—C11	109.89 (11)
C2—C1—H1	129.6 (9)	C10—C8—C11	112.67 (12)
O1—C2—N3	122.08 (12)	O3—C8—C9	101.68 (11)
O1—C2—C1	120.21 (13)	C10—C8—C9	111.04 (12)
N3—C2—C1	117.64 (12)	C11—C8—C9	110.43 (13)
N3—C3—C5	110.49 (12)	C8—C9—H91	109.5
N3—C3—H31	109.6	C8—C9—H92	109.5
C5—C3—H31	109.6	H91—C9—H92	109.5
N3—C3—H32	109.6	C8—C9—H93	109.5
C5—C3—H32	109.6	H91—C9—H93	109.5
H31—C3—H32	108.1	H92—C9—H93	109.5
N3—C4—C6	110.29 (12)	C8—C10—H101	109.5
N3—C4—H41	109.6	C8—C10—H102	109.5
C6—C4—H41	109.6	H101—C10—H102	109.5
N3—C4—H42	109.6	C8—C10—H103	109.5
C6—C4—H42	109.6	H101—C10—H103	109.5
H41—C4—H42	108.1	H102—C10—H103	109.5
N4—C5—C3	109.92 (12)	C8—C11—H111	109.5
N4—C5—H51	109.7	C8—C11—H112	109.5
C3—C5—H51	109.7	H111—C11—H112	109.5
N4—C5—H52	109.7	C8—C11—H113	109.5
C3—C5—H52	109.7	H111—C11—H113	109.5
H51—C5—H52	108.2	H112—C11—H113	109.5
N4—C6—C4	110.27 (12)		
			
C4—N3—C2—O1	−4.1 (2)	C7—N4—C6—C4	128.14 (15)
C3—N3—C2—O1	−165.84 (14)	C5—N4—C6—C4	−57.04 (17)
C4—N3—C2—C1	178.83 (13)	N3—C4—C6—N4	53.39 (17)
C3—N3—C2—C1	17.1 (2)	C8—O3—C7—O2	2.4 (2)
N2—C1—C2—O1	−3.6 (2)	C8—O3—C7—N4	−178.51 (11)
N2—C1—C2—N3	173.55 (13)	C5—N4—C7—O2	−177.86 (14)
C2—N3—C3—C5	−143.07 (14)	C6—N4—C7—O2	−3.7 (2)
C4—N3—C3—C5	54.32 (16)	C5—N4—C7—O3	3.0 (2)
C2—N3—C4—C6	142.52 (13)	C6—N4—C7—O3	177.17 (13)
C3—N3—C4—C6	−53.89 (17)	C7—O3—C8—C10	62.39 (16)
C7—N4—C5—C3	−128.35 (15)	C7—O3—C8—C11	−62.62 (16)
C6—N4—C5—C3	57.18 (17)	C7—O3—C8—C9	−179.61 (12)
N3—C3—C5—N4	−53.86 (17)		

### Hydrogen-bond geometry (Å, °)

**Table:**

*D*—H···*A*	*D*—H	H···*A*	*D*···*A*	*D*—H···*A*
C1—H1···O1^i^	0.946 (17)	2.313 (18)	3.250 (3)	170.6 (14)
C3—H32···O1^i^	0.99	2.36	3.327 (3)	164

Symmetry codes: (i) −*x*, *y*+1/2, −*z*+1/2.
